# Non-*Helicobacter pylori Helicobacter* Species as a Cause of Refractory Chronic Cellulitis in X-Linked Agammaglobulinemia

**DOI:** 10.1007/s10875-024-01668-y

**Published:** 2024-02-16

**Authors:** Qianqian Zhao, Jijun Ma, Jiawen Wu, Abdurahman Matruzi, Chongwei Li

**Affiliations:** 1 Department of Rheumatology & Clinical Immunology, Tianjin Children’s Hospital, Tianjin, China; 2 Tianjin Key Laboratory of Birth Defects for Prevention and Treatment, Tianjin, China; 3 Department of Internal Medicine, Tianjin Children’s Hospital, Tianjin, China

**To the Editor,**

X-linked agammaglobulinemia (XLA) is an inborn error of immunity (IEI) first described in 1952 and has a prevalence of 1:100,000 to 1:200,000 [1]. A loss-of-function mutation in Bruton’s tyrosine kinase (BTK) gene results in B-cell maturation failure and defective antibody production. XLA is generally characterized by recurrent infections, agammaglobulinemia, and a percentage of circulating CD19^+^ B cells less than 2%. Sinopulmonary infections are common in XLA, while skin infections are also significant. Non*-Helicobacter pylori Helicobacter* (NHPH) species, representing a rare cause of chronic conditions in XLA patients, are challenging to detect and eradicate [2]. In this paper, we aim to explain the characteristic features of NHPH infections in XLA patients. Moreover, we present a rational and efficient method for diagnosing and treating these formidable infections.

## Case Description

**Fig. 1:**
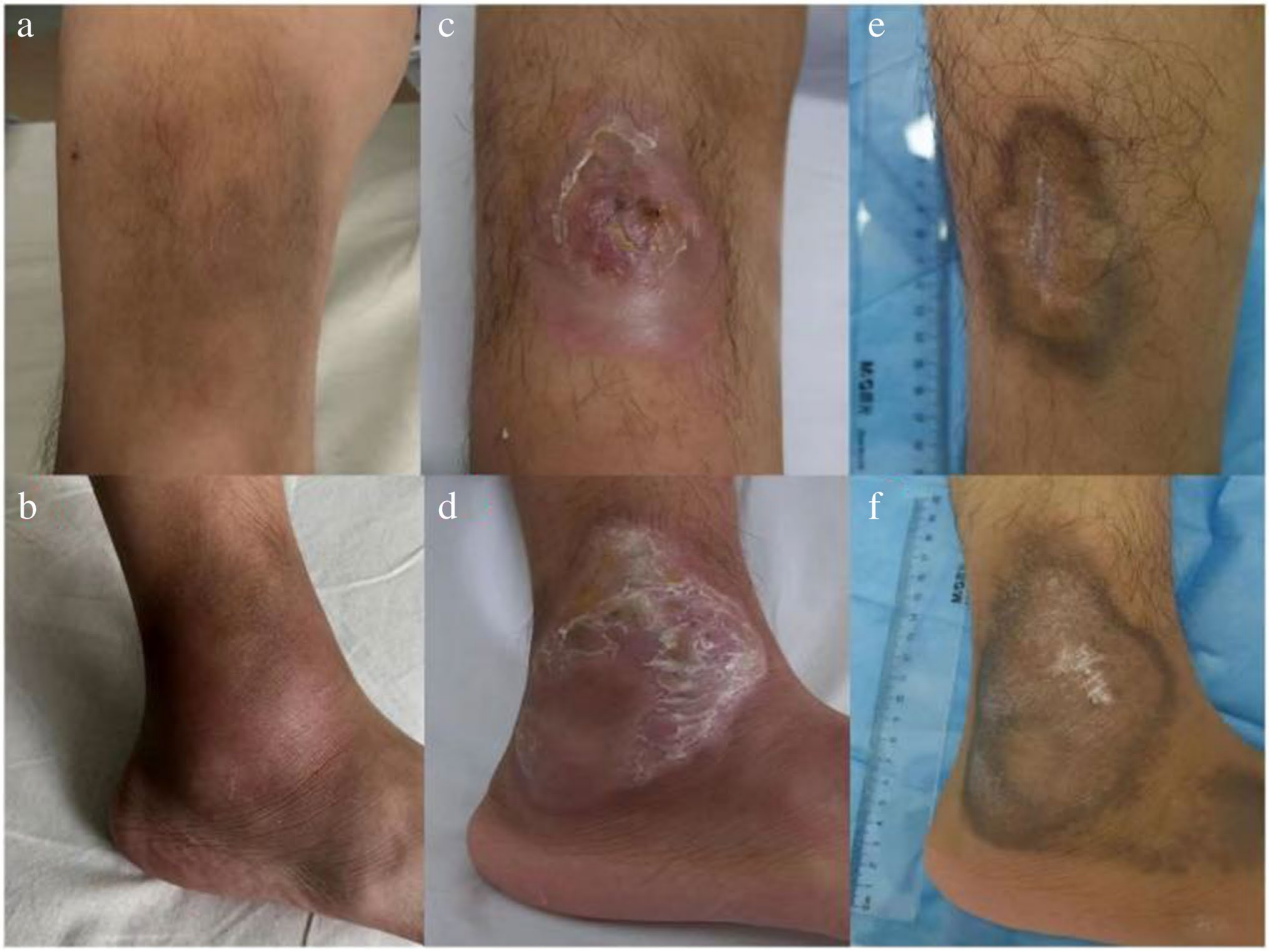
Chronic cellulitis on the lower extremities (right) of the patient with XLA. **a, b** At presentation (aged 14 years), cellulitis lesions with demarcated, hyperpigmented, painful, macule, indurated plaques on the lower extremities (right). **c, d** Before combining antibiotics of imipenem and fosfomycin treatment (aged 17 years), cellulitis PG-like ulcers on the lower extremities (right). **e, f** After 10-week systemic antibiotics treatment (aged 17 years), cellulitis lesions healed; only pigmentation remained on the lower extremities (right)

A 17-year-old boy was admitted to our Department due to a 5-year history of skin infections. He lived in a rural area, and his family raised pigs in the yard. There was no family history of immunodeficiency. He developed perianal abscess at 3 months of age, suffered from septicaemia caused by *Pseudomonas aeruginosa* infection at 16 months of age and cutaneous abscess at 22 months of age. At the time of examination, no tonsillar tissue was present. Immunological evaluation revealed extremely low levels of IgG (10 mg/dl), IgA (2 mg/dl), IgM (0 mg/dL), and CD19^+^ circulating B cells (0%). Whole-exome sequencing revealed a frameshift mutation (1581-1584de1TTTG) in exon 16 of the BTK gene (p.Cys527trpfsX2). He was on irregular replacement therapy with intravenous immunoglobulin (IVIG) (trough IgG level = 30–143 mg/dL). At 12 years of age, he had demarcated, hyperpigmented, painful, macular, and indurated plaques on his right ankle extensor surface accompanied by an irregular fever. By 14 years of age, the lesion had spread from the right ankle to the tibial regions of both legs (Fig. 1a, b). Monthly IVIG replacement (500–800 mg/kg; trough IgG level = 600–800 mg/dL) was initiated; however, the lower extremity lesions persisted. At 17 years of age, the lower extremity cellulitis worsened, with pyoderma gangrenosum (PG)-like leg ulcers (Fig. 1c, d) and lymphangitis in the right lower extremity accompanied by recurrent moderate-to-high fever. Routine blood tests revealed the following: white blood cell count, 6.5–14.83 × 10^9^/L; 75–82% neutrophils, and 10–15% lymphocytes. Other laboratory results were normal, except for an elevated CRP level (12.67–70.99 mg/L, normal value = 0–8 mg/L). Previous conventional blood and pus cultures were negative. The patient’s guardians declined skin biopsy. The lesions responded well to cefoperazone/sulbactam and linezolid combined treatment. Nevertheless, relapse episodes occurred following antibiotic treatment cessation, and the patient eventually developed cefoperazone-sulbactam sodium and linezolid resistance during prolonged therapy. He was treated with cefepime, vancomycin, amoxicillin, amikacin, levofloxacin, and ertapenem successively; however, all of these antibiotics demonstrated transient efficacy or inefficacy. Two months later, incision and drainage were performed, revealing chocolate-colored pus. Metagenomic next-generation sequencing (mNGS) was also conducted to determine the causative agent. *H. equorum* and *H. canadensis* were identified in both blood and pus. Subsequently, the patient was treated with intravenous fosfomycin sodium (4 g, every 8 hours) combined with imipenem-cilastatin sodium (0.5 g, every 6 hours). He was afebrile and showed improvement in his cellulitis on the second day, and his CRP level normalized rapidly. Simultaneously, the boy presented with neutropenia (1.07×10^9^/L). Additionally, the patient displayed hypersthenuria (urine-specific gravity ≥1.037), urobilinogenuria (urobilinogen 3+), and proteinuria (urine protein 1+) while maintaining normal renal function. After 10 weeks of intravenous antibiotic treatment, the PG-like ulcer resolved, with only pigmentation remaining (Fig. 1e, f). The antibiotics doxycycline (100 mg twice daily) and rifampin (450 mg once daily) were orally administered for the next ten weeks. The patient was stable after oral maintenance therapy, and his complete blood count and urine analysis gradually normalized within 2 months after antibiotic discontinuation. The 15-month posttherapy follow-up showed no cellulitis recurrence.

## Discussion

*Helicobacter* is a genus of gram-negative bacteria characterized by their spiral shape and microaerophilic nature. NHPH species are zoonotic and may be linked to exposure to pigs in our case [2]. The most clinically significant NHPH species are *H. cinaedi* and *H. bilis*. However, sporadic cases of *H. equorum* and *H. canadensis* have also been reported [3, 4]. Many different symptoms are caused by these species, including recurrent fevers, abdominal abscesses, bacteraemia, osteomyelitis, arthritis, cellulitis, and PG-like ulcers. Culture is a traditional method with time-consuming and lowest positive rate due to the fastidious nature of NHPH. Most cases are identified by 16S sequencing, which is faster and more accurate than culture; however, as with other approaches (including polymerase chain reaction and mass spectrometry), accessibility is a major problem that hinders its clinical use. Given that mNGS assays for a diverse range of agents in an unbiased manner, it has the potential to enhance the detection of pathogens that may have been missed by clinicians or previously methods. Our case study highlights the significance of mNGS as a rapid, noninvasive, and valuable tool. The most important drawback of mNGS is the inability to identify antibiotic susceptibility.

Moreover, our case study reveals distinctive indications of infections with *H. equorum* and *H. canadensis* in XLA patients [4]. Specifically, the lower extremities are frequently affected, with lesions initially appearing as hyperpigmented and indurated areas that progress over months to years to form well-defined, painful macules, or chronic ulcers. Local factors (trauma) may contribute to the persistence of lesions on the lower extremities compared with the upper extremities. Individuals with XLA are prone to a proinflammatory state, rendering them susceptible to developing PG or PG-like lesions [2]. Characteristic skin lesions are significant clinical indicators in patients with disseminated NHPH infections. More clinical manifestations of XLA with NHPH infections are available in Table S1.

NHPH species are predominantly extracellular bacteria with extensive genetic mechanisms to escape host defenses, which contribute to their ability to persist. They are resistant to multiple antibiotic classes. For example, *H. canadiensi* and its isolates are resistant to azithromycin, ampicillin, clarithromycin, levofloxacin, and ciprofloxacin [2]. Unfortunately, there is no consensus treatment for NHPH infections. Overall, the antibiotics reported to be effective at eradicating this infection are broad-spectrum antibiotics, including a combination of two or more antibiotics. Our case showed that intravenous fosfomycin sodium combined with imipenem cilastatin sodium and sequential doxycycline combined with rifampicin were effective. A summary of antibiotics regimens used (failed and successful with durations) is available in Table S2. Our patient was claustrophobic and did not tolerate hyperbaric oxygen therapy (HBO); however, HBO may be a potential adjunctive therapy for patients with refractory chronic cellulitis for it alleviates inflammation, maintains wound oxygenation, and stimulates wound repair. NHPH infections are rare but significant causes of cellulitis in XLA patients. The lack of IgA in XLA patients may contribute to their susceptibility to disseminated infections. Consequently, adequate IVIG treatment and normal serum IgG levels do not appear to provide complete protection against NHPH infections in XLA patients [2, 5].

In conclusion, NHPH infections should be considered when cellulitis is refractory in patients with XLA, even if IgG levels are normal. NHPH species are challenging to identify using conventional microbiological techniques. Therefore, mNGS may be crucial for detecting these elusive microorganisms. Given the risk of recurrence, a combination of antibiotics and prolonged duration of therapy may be required for effective management, along with drug toxicity monitoring and close follow-up.

### Supplementary Information

ESM 1 (DOCX 35 kb) (PDF 52 kb)

ESM 2 (DOCX 22 kb) (PDF 110 kb)

ESM 3 (PDF 33 kb)

ESM 4 (PDF 53 kb)

## Data Availability

No datasets were generated or analyzed during the current study.
