# Supplementary material for: Non-Helicobacter pylori Helicobacter Species as a Cause of Refractory Chronic Cellulitis in X-Linked Agammaglobulinemia
Source: J Clin Immunol. 2024 Feb 16;44(3):65. doi: 10.1007/s10875-024-01668-y (PMC10873429; doi:10.1007/s10875-024-01668-y)
Supplement: Supplementary file 1 — (DOCX 35 kb) (PDF 52 kb) [file 10875_2024_1668_MOESM1_ESM.pdf]

Journal Name: Journal of Clinical Immunology

Title: Non-Helicobacter pylori Helicobacter species as a cause of refractory chronic Cellulitis in X-linked agammaglobulinemia

Qianqian Zhao[1], Jijun Ma1, Jiawen Wu[1], Abdurahman•Matruzi[2], Chongwei Li[1]*

1 Department of Rheumatology & Clinical Immunology, Tianjin Children's Hospital (Tianjin University Children's Hospital), Tianjin, China; Tianjin Key Laboratory of Birth Defects for Prevention and Treatment, Tianjin, China

2 Department of Internal Medicine, Tianjin Children's Hospital (Tianjin University Children's Hospital), Tianjin, China

* Correspondence: leechongwei@126.com

Library preparation and metagenomic sequencing

DNA library was prepared by automatic nucleic acid extraction, enzymatic fragmentation, end repair, terminal adenylation and adaptor ligation. Finished libraries were quantified by real-time PCR (KAPA) and pooled. Shotgun sequencing was carried out on illumina Nextseq. Approximately 20 million of 50bp single-end reads were generated for each library. Bioinformatic analysis was conducted as described in a previous report. Briefly, sequences of human origin were filtered (GRCh38.p13) and the remaining reads were aligned to a reference database (NCBI nt, GenBank and in-house curated genomic database) to identify the microbial species and read count. For each sequencing run, a negative control (culture medium containing 104 Jurkat cells/ml) was included.

mNGS reporting criteria

Microbial reads identified from a library were reported if: 1) the sequencing data passed quality control filters (library concentration > 50 pM, Q20 > 85%, Q30 > 80%); 2) negative control (NC) in the same sequencing run does not contain the species or the RPM (sample) / RPM (NC) ≥ 5, which was determined empirically according to previous studies as a cutoff for discriminating true-positives from background contaminations
