# Supplementary material for: Non-Helicobacter pylori Helicobacter Species as a Cause of Refractory Chronic Cellulitis in X-Linked Agammaglobulinemia
Source: J Clin Immunol. 2024 Feb 16;44(3):65. doi: 10.1007/s10875-024-01668-y (PMC10873429; doi:10.1007/s10875-024-01668-y)
Supplement: Supplementary file 2 — (DOCX 22 kb) (PDF 110 kb) [file 10875_2024_1668_MOESM2_ESM.pdf]

TABLE 1　Summary of case reports of NHPH infections in XLA（Total 27cases）.

			Cases	（%）			Sample	Cases	（%）
Species	single pathogen	H. Cinaedi	11	40.74	Diagnosis method	16S sequencing （14cases, 51.85%）	Blood	2	7.41
		H. bilis	7	25.92			Biopsy	6	22.22
		H.canis	1	3.70			Pus	1	3.70
		H. fennelliae	1	3.70			Blood cultured bottles	5	18.52
		Helicobacter sp	1	3.70		PCR	-	3	11.11
		Helicobacter/Flexispira-like organism	1	3.70		mNGS（*）	Blood（*）	2	7.41
		H. Equorum -like	1	3.70			Pus（*）	2	7.41
		Canadensis-like	1	3.70		Cultures	Blood	4	14.81
		Helicobacter trogontum	1	3.70			Pus	0	0.00
	coinfection	H. Canis and H. Bilis （F. rappini）	1	3.70		23S sequencing	-	1	3.70
		H.equorum and H.canadensis（*）	1	3.70		Mass spectrometry	-	5	18.52
Type of infection	cellulitis（*） （17cases, 62.96%）	Lower limbs（*）	15	55.55	Antibiotics	Imipenem/cilastin （*）	-	8	29.63
		upper limb	4	14.81		Meropenem	-	5	18.52
		unknown	2	7.41		Ertapenem	-	3	11.11
	P-G-like ulcer（*） （7cases, 25.92%）	lower limbs（*）	6	22.22		Panipenem/betamipron	-	1	3.70
		upper limbs	1	3.70		Ceftazidime	-	1	3.70

TABLE 1 continue

	Hyperpigmented plaques and subcutaneous nodules	both legs	1	3.70		Doxycycline （*）	-	7	25.93
	Erythematous plaques	ankle	1	3.70		Minocycline	-	6	22.22
	* skin infection （all the above）	-	19	70.37		Amoxicillin/amoxicillinclavulanate potassium	-	4	14.81
		-				Netronidazole	-	4	14.81
	Sepsis（*）	-	12	44.44		Ciprofloxacin	-	1	3.70
	Osteomyelitis	-	2	7.41		Tobramycin	-	1	3.70
	Suppurative cholangitis	-	3	11.11		Rifampicin （*）	-	3	11.11
Type of infection	Abdominal abscess	-	1	3.70	Antibiotics	Clarithromycin	-	3	11.11
	Refractory chronic pleurisy	-	1	3.70		Levofloxacin	-	1	3.70
	synovitis	-	1	3.70		Amikacin	-	1	3.70
						Moxifloxacin	-	2	7.41
						Gentamicin	-	5	18.52
						Ceftriaxone	-	3	11.11
						Cefepime	-	1	3.70
						TrimethoprimSulfamethoxazole	-	2	7.41
						Vancomycin	-	1	3.70
						Linezolid	-	1	3.70
						Kanamycin	-	1	3.70
						Fosfomycin（*）	-	2	7.41
						Azithromycin	-	2	7.41

（*）：our patient * There are 4 patients have both cellulitis and PG-like ulcers, and 4patients have both upper and lower extremity cellulitis.

References

[1]Weir S, Cuccherini B, Whitney AM, et al. Recurrent Bacteremia Caused by a “Flexispira”-Like Organism in a Patient With X-Linked (Bruton’ s) Agammaglobulinemia. J. Clin.Microbiol. 1999;37, 2439 – 2445.

[2] Cuccherini B, Chua K, Gill V, et al.Bacteremia and Skin/Bone Infections in Two Patients With X-Linked Agammaglobulinemia Caused by an Unusual Organism Related to Flexispira/Helicobacter Species. Clin. Immunol. 2000;97, 121-129.

[3]Han S, Schindel C., Genitsariotis R, et al. Identification of a Unique Helicobacter Species by 16S rRNA Gene Analysis in an Abdominal Abscess From a Patient With X-Linked Hypogammaglobulinemia. J. Clin. Microbiol. 2000;38, 2740 – 2742.

[4]Gerrard J, Alfredson D, and Smith I. Recurrent Bacteremia and Multifocal Lower Limb Cellulitis Due to Helicobacter-Like Organisms in a Patient With X-Linked Hypogammaglobulinemia. Clin. Infect. Dis. 2001,33, E116 – E118.

[5]Simons E, Spacek L A, Lederman HM, and Winkelstein J A. Helicobacter Cinaedi Bacteremia Presenting as Macules in an Afebrile Patient With X-Linked Agammaglobulinemia. Infection;2004,32, 367 – 368.

[6]Leemann C, Gambillara E, Prod'hom G ,et al.First case of bacteremia and multifocal cellulitis due to Helicobacter canis in an immunocompetent patient.J Clin Microbiol.2006 ;44(12):4598-600.

[7] Schwarze-Zander C, Becker S, Wenzel J,et al. Bacteremia Caused by a Novel Helicobacter Species in a 28-Year-Old Man Wi[th X-Linked Agammaglobulinemia. J. Clin. Microbiol. 2010;48,4672 – 4676.

[8]Murray P R, A Uzel, G Ranken, et al. Pyoderma Gangrenosum-Like Ulcer in a Patient With X-Linked Agammaglobulinemia: Identification of Helicobacter Bilis by Mass Spectrometry Analysis. Arch.2010;146, 523–526. doi: 10.1001/archdermatol.2010.86

[9]Funato M, Kaneko H, Ohkusu K, et al.. Refractory Chronic Pleurisy Caused by Helicobacter Equorum-Like Bacterium in a Patient With X-Linked Agammaglobulinemia. J. Clin.Microbiol. 2011;49, 3432 – 3435.

[10]Sharp S E. Chronic Skin Lesions From a Patient With Bruton’s X-Linked Agammaglobulinemia. J. Clin. Microbiol. 2011;49, 483 – 48+.

[11]Dua J, Elliot E, Bright P, et al.Pyoderma Gangrenosum-Like Ulcer Caused by Helicobacter Cinaedi in a Patient With X-Linked Agammaglobulinaemia. Clin. Exp. Dermatol. 2012;37, 642 – 645

[12]Turvey SE, Leo SH, Boos A, et al. Successful Approach to Treatment of Helicobacter Bilis Infection in X Linked Agammaglobulinemia. J. Clin. Immunol. 2012;32, 1404 – 1408.

[13]Toyofuku M, Tomida J., Kawamura Y,et al. Helicobacter Cinaedi Bacteremia Resulting From Antimicrobial Resistance Acquired During Treatment for X-Linked Agammaglobulinemia.Infect. Chemother. 2016;22, 704 – 706.

[14]Degand N, Dautremer J, Pilmis B, et al. Helicobacter Bilis-Associated Suppurative Cholangitis in a Patient With X-Linked Agammaglobulinemia. J. Clin. Immunol. 2017;37, 727 – 731.

[15]Hill A, Byrne A, Bouffard D,et al. Helicobacter Cinaedi Bacteremia Mimicking Eosinophilic Fasciitis in a Patient With X-Linked Agammaglobulinemia. JAAD Case Rep. 2018;4, 327 – 329.

[16]Matsumoto A, Yeh I, Schwartz B,et al. Chronic Helicobacter Cinaedi Cellulitis Diagnosed by Microbial PolymeraseChain Reaction,2017; JAAD Case Rep. 3, 398 – 400.

[17]Sugimoto M, Takeichi T, Muramatsu H, et al. Recurrent Cellulitis Caused by Helicobacter Cinaedi in a Patient With X-Linked Agammaglobulinaemia. Acta Derm. Venereol. 2017;97, 277 – 278.

[18]Inoue K, Sasaki S, Yasumi T, et al. Helicobacter Cinaedi-Associated Refractory Cellulitis in Patients With XLinked Agammaglobulinemia. J Clin Immunol 2020;40, 1132 – 1137.

[19]Fettinger NB, Bradley, JS, Ramchandar N. An 11-Year-old Male With X-linked Agammaglobulinemia and Persistent Abdominal Pain.Pediatr Infect Dis J.2021;1;40(4):356 -358

[20]Romo-Gonzalez C , Bustamante-Ogando JC, Yamazaki-Nakashimada MA,et al.Infections With Enterohepatic Non- H. pylori Helicobacter Species in X-Linked Agammaglobulinemia: Clinical Cases and Review of the Literature.Front Cell Infect Microbiol. 2022;4:11:807136.

[21]Lenskaya V , O'Connor M,de Moll EH,et al.Relapsing Helicobacter cinaedi Cellulitis without Bacteremia in a Patient with X-linked Agammaglobulinemia after 1 Year of Antibiotic Prophylaxis.Skinme. 2022 ;20;20(6):457-459.

[22]Fjordside L, Herløv C ,Drabe CH,et al.Helicobacter trogontum Bacteremia and Lower Limb SkinLesion in a Patient with X-Linked Agammaglobulinemia-ACase Report and Review of the Literature.Pathogens.2022 ;27;11(11):1247.

[23]Zhang L, Zhou ML, Lv W,et al.Metagenomics assists in the diagnosis of a refractory, culture-negative pyoderma gangrenosum-like ulcer caused by Helicobacter cinaedi in a patient with primary agammaglobulinemia.J Microbiol Immunol Infect.2023;56(6):1284-1287.
