# Supplementary material for: Non-Helicobacter pylori Helicobacter Species as a Cause of Refractory Chronic Cellulitis in X-Linked Agammaglobulinemia
Source: J Clin Immunol. 2024 Feb 16;44(3):65. doi: 10.1007/s10875-024-01668-y (PMC10873429; doi:10.1007/s10875-024-01668-y)
Supplement: Supplementary file 3 — (PDF 33 kb) [file 10875_2024_1668_MOESM3_ESM.pdf]

SPRINGER NATURE

Author Services

# Editing Certificate

This document certifies that the manuscript

## Non-Helicobacter pylori Helicobacter species as a cause of refractory chronic Cellulitis in X-Linked Agammaglobulinemia

prepared by the authors

Qianqian Zhao, Jijun Ma, Jiawen Wu, Abdurahman·Matruzi, Chongwei Li

was edited for proper English language, grammar, punctuation, spelling, and overall style by one or more of the highly qualified native English speaking editors at SNAS.

This certificate was issued on **January 30, 2024** and may be verified on the SNAS website using the verification code **EBC9-6CA5-C56B-7248-B6AP**.

Neither the research content nor the authors' intentions were altered in any way during the editing process. Documents receiving this certification should be English-ready for publication; however, the author has the ability to accept or reject our suggestions and changes. To verify the final SNAS edited version, please visit our verification page at secure.authorservices.springernature.com/certificate/verify.
If you have any questions or concerns about this edited document, please contact SNAS at support@as.springernature.com.

SNAS provides a range of editing, translation, and manuscript services for researchers and publishers around the world.
For more information about our company, services, and partner discounts, please visit authorservices.springernature.com.
