# Supplementary material for: Non-Helicobacter pylori Helicobacter Species as a Cause of Refractory Chronic Cellulitis in X-Linked Agammaglobulinemia
Source: J Clin Immunol. 2024 Feb 16;44(3):65. doi: 10.1007/s10875-024-01668-y (PMC10873429; doi:10.1007/s10875-024-01668-y)
Supplement: Supplementary file 4 — (PDF 53 kb) [file 10875_2024_1668_MOESM4_ESM.pdf]

Journal Name: Journal of Clinical Immunology

Title: Non-Helicobacter pylori Helicobacter species as a cause of refractory chronic Cellulitis in X-linked agammaglobulinemia

Qianqian Zhao1, Jijun Ma1, Jiawen Wu1, Abdurahman•Matruzi2, Chongwei Li1*

1 Department of Rheumatology & Clinical Immunology, Tianjin Children's Hospital (Tianjin University Children's Hospital), Tianjin, China; Tianjin Key Laboratory of Birth Defects for Prevention and Treatment, Tianjin, China

2 Department of Internal Medicine, Tianjin Children's Hospital (Tianjin University Children's Hospital), Tianjin, China

* Correspondence: leechongwei@126.com

TABLE2 Antibiotics regimens used

Time	Regimen	Duration	Effect
2019.1~2020.10	ceftriaxone	1 week	transient
	latamoxef	1 week	transient
	mezlocillin/sulbactam+linezolid(P.O)	1 week	transient
	levofloxacin	1 week	failed
	moxifloxacin	1 week	failed
	cefdinir	1 week	failed
	cefuroxime axetil	1 week	failed
	cefoperazone-sulbactam sodium(monotherapy)	2 weeks	effective
	cefoperazone-sulbactam sodium+linezoli(P.O)	2 weeks	effective
	cefoperazone-sulbactam sodium+vancomycin	2 weeks	effective
2021.9.6	cefoperazone-sulbactam sodium+linezolid(P.O)	2 weeks	effective
2021.12.30	cefoperazone-sulbactam sodium+vancomycin	2 weeks	effective
2022.1.25~2.22	cefoperazone-sulbactam sodium+doxycycline(I.V)	2 weeks	effective/transient
	cefepime	3 days	failed
	fosfomycin sodium+ertapenem	5 days	transient
	amoxicillin(P.O) +amikacin	1 weeks	transient
2022.2.23~3.22	amoxicillin(P.O)+amikacin+rifampin	2 weeks	effective/transient
	cefoperazone-sulbactam	2 weeks	effective/transient

TABLE 2 continue

	sodium+doxycycline(I.V)		
2022.3.22~4.21	cefoperazone-sulbactam sodium+doxycycline(P.O)+rifampin	10 days	effective/transient
	amikacin+doxycycline(P.O)+rifampin	10 days	effective/transient
	cefoperazone-sulbactam sodium+doxycycline(I.V)	10 days	effective/transient
2022.4.22~5.22	metronidazole+amoxicillin +clindamycin	4 weeks	failed
2022.5.23~5.26	cefoperazone-sulbactam sodium	4 days	failed
2022.5.27~8.5	fosfomycin sodium+imipenem-cilastatin sodium	10 weeks	successful
2022.8.6~10.15	doxycycline+rifampin	10 weeks	successful
